# Coregistered histology sections with diffusion tensor imaging data at 200 µm resolution in meningioma tumors

**DOI:** 10.1016/j.dib.2023.109261

**Published:** 2023-05-23

**Authors:** Jan Brabec, Elisabet Englund, Johan Bengzon, Filip Szczepankiewicz, Danielle van Westen, Pia C. Sundgren, Markus Nilsson

**Affiliations:** a Medical Radiation Physics, Clinical Sciences Lund, Lund University, Lund, Sweden; b Russell H. Morgan Department of Radiology and Radiological Science, Johns Hopkins University School of Medicine, Baltimore, MD, USA; c M. Kirby Research Center for Functional Brain Imaging, Kennedy Krieger Institute, Baltimore, MD, USA; d Pathology, Clinical Sciences, Lund University, Lund, Sweden; e Division of Neurosurgery, Skane University Hospital, Lund, Sweden; f Stem Cell Center, Department of Clinical Sciences, Lund, Sweden; g Diagnostic Radiology, Clinical Sciences Lund, Lund University, Lund, Sweden; h Lund University Bioimaging Centre, Lund University, Lund, Sweden; i Department of Medical Imaging and Physiology, Skåne University Hospital, Lund University, Lund, Sweden

**Keywords:** Mean diffusivity, Fractional anisotropy, Diffusion tensor imaging, Meningioma, Microstructure, Coregistration, Hematoxylin & Eosin, Elastica van Gieson

## Abstract

A significant problem in diffusion MRI (dMRI) is the lack of understanding regarding which microstructural features account for the variability in the diffusion tensor imaging (DTI) parameters observed in meningioma tumors. A common assumption is that mean diffusivity (MD) and fractional anisotropy (FA) from DTI are inversely proportional to cell density and proportional to tissue anisotropy, respectively. Although these associations have been established across a wide range of tumors, they have been challenged for interpreting within-tumor variations where several additional microstructural features have been suggested as contributing to MD and FA.

To facilitate the investigation of the biological underpinnings of DTI parameters, we performed ex-vivo DTI at 200 µm isotropic resolution on sixteen excised meningioma tumor samples. The samples exhibit a variety of microstructural features because the dataset includes meningiomas of six different meningioma types and two different grades. Diffusion-weighted signal (DWI) maps, DWI maps averaged over all directions for given b-value, signal intensities without diffusion encoding (S_0_) as well as DTI parameters: MD, FA, in-plane FA (FA_IP_), axial diffusivity (AD) and radial diffusivity (RD), were coregistered to Hematoxylin & Eosin- (H&E) and Elastica van Gieson-stained (EVG) histological sections by a non-linear landmark-based approach.

Here, we provide DWI signal and DTI maps coregistered to histology sections and describe the pipeline for processing the raw DTI data and the coregistration. The raw, processed, and coregistered data are hosted by Analytic Imaging Diagnostics Arena (AIDA) data hub registry, and software tools for processing are provided via GitHub. We hope that data can be used in research and education concerning the link between the meningioma microstructure and parameters obtained by DTI.

**Specifications Table**

**Table:** 

Subject	Health and medical sciences: Medical Imaging.
Specific subject area	Diffusion magnetic resonance imaging and histopathological sections.
Type of data	Diffusion-weighted signal and digitalized histology sections.
How the data were acquired	Bruker 9.4 T BioSpec Avance III MRI scanner. Microscope slide scanner Hamamatsu NanoZoomer S360. The data were further processed with an in-house diffusion tensor imaging pipeline and registration tool.
Data format	Raw and analyzed data.
Description of data collection	The dataset includes tumor samples from sixteen patients that were diagnosed with meningioma tumors and scheduled for surgical treatment between 2016 and 2018 at Skåne University Hospital, Lund, Sweden. Inclusion criteria were age above 18 years, histologically confirmed meningioma, and signed informed consent.
Data source location	Patients: Neurosurgery, Clinical Sciences, Lund University, Lund, Sweden, and Diagnostic Radiology, Clinical Sciences, Lund University, Lund, Sweden. DTI measurements: Lund University Bioimaging Centre (LBIC), Lund University, Lund, Sweden. Histology slides: Pathology, Clinical Sciences, Lund University, Lund, Sweden.
Data accessibility	Dataset name of the raw and processed data: MicroMen at Analytic Imaging Diagnostics Arena (AIDA) data hub registry. Data identification number: 10.23698/aida/micromen. Direct URL to data: https://www.doi.org/10.23698/aida/micromen. Instructions for accessing the software: The data are distributed under AIDA BY license: https://datahub.aida.scilifelab.se/10.23698/aida/micromen#aida-by-license Repository name of the software processing tools: Microimaging vs histology meningiomas DIB. Direct URL to repository: https://github.com/jan-brabec/microimaging_histology_DIB. Instructions for accessing the software: The processing tools are distributed under CC BY-NC-SA 4.0 license: https://creativecommons.org/licenses/by-nc-sa/4.0/
Related research article	Brabec, J., Friedjungová, M., Vašata, D., Englund, E., Bengzon, J., Knutsson, L., Szczepankiewicz, F., van Westen, D., Sundgren, P.C. and Nilsson, M., 2023. Meningioma microstructure assessed by diffusion MRI: An investigation of the source of mean diffusivity and fractional anisotropy by quantitative histology. NeuroImage: Clinical, 37, p.103365. Direct URL to related research article: https://doi.org/10.1016/j.nicl.2023.103365 Direct URL to the repository of the related research article: https://github.com/jan-brabec/microimaging_vs_histology_in_meningeomas.

## Value of the Data

• The data are useful to understand what histopathological features explain the variability in DTI measurements.
• The data can be used to further identify features of relevance and quantify their impact on the DTI parameters.
• As high-resolution DTI measurements as well as voxel-to-voxel coregistration with histology are costly and time-consuming, only a few DTI-histology correlation datasets have been published.
• The data can also be useful for those aiming to understand how histopathological images are connected to WHO type and grade.
• Data might also be useful for improving coregistration techniques between MR images and histology sections or between different staining of histology sections.
• Results are of value for those aiming at studying the difference in histological appearance of meningioma subtypes or for educational purposes.

## 1 Objective

Diffusion MRI (dMRI) is the primary modality for obtaining information on tumor microstructure non-invasively [1,2]. Diffusion tensor imaging (DTI) is widely applied in patients with intracranial tumors and yields several key parameters: the mean diffusivity (MD), the fractional anisotropy (FA), axial diffusivity (AD), and radial diffusivity (RD) [3]. The central question is what microstructural features impact the DTI measurements and map onto these DTI parameters in tumors. It has been found that MD correlates negatively with cell density in a wide range of tumor types [4,5]. Furthermore, the FA may reflect the voxel-level diffusion anisotropy [6,7]. However, other features may be also relevant. As for MD, these may include cell size [8], membrane permeability [9], the presence of necrosis [10], or large interstitial spaces within the tumor stroma [11,12]. These can also include microcysts, tumor vasculature, psammoma bodies, or tissue cohesivity [13]. Furthermore, FA reflects macroscopic (voxel-level) anisotropy, which is lower than the microscopic diffusion anisotropy due to the presence of orientation dispersion [14]. In summary, DTI measurements, i.e. DWI signal values, as well as DTI parameters, such as MD, FA, in-plane FA, AD, and RD, are affected by a multitude of features and their impact within meningioma tumors has not yet been quantified in detail. Therefore, we provide a dataset with high-resolution DTI data (200 µm) coregistered with histology sections stained with Hematoxylin and Eosin stain (H&E) and Elastica van Gieson stain (EVG).

The data can be used to identify the histological features that contribute to DTI measurements at the microscopic level. For example, the data can be used to study which histological features are of relevance to explain the variability or to predict the outcome of the DTI measurements. They may also be used to explore the inverse task, i.e. the class of features mapping onto similar DTI measurement values. Our related research article qualitative analysis suggested some features of relevance for DTI such as the presence of assembled blood vessels, microcysts, and psammoma bodies [13]. Future work should preferably examine the quantitative basis of this finding by devising a method to compute the extent of these features from the histology images.

## 2 Data Description

The dataset from the AIDA repository is the coregistered histology sections with DTI. Table 1 lists all sixteen samples and their histopathological classification based on the WHO criteria [15]. Table 2 describes the diffusion-weighted imaging protocol applied to each meningioma tumor sample (diffusion encoding direction, prescribed and effective b-values), and Table 3 the data folder structure related to the DTI measurement prior to the coregistration. Table 4 describes the data structure for the coregistered images for each sample s (s = 1, 2,… 16) and the data structure is the same for all sixteen individual samples. The following DTI parameters were obtained: mean diffusivity (MD), fractional anisotropy (FA), in-plane fractional anisotropy (FA_IP_), which is further described in [13], axial diffusivity (AD), radial diffusivity (RD). We also coregistered DWI data without diffusion encoding (S_0_ map), all DWI data, averaged DWI data across directions for given b-value, and diffusion tensor eigenvalues in the x-y plane (J_11_, J_12_, J_22_). The code for processing the data is available and is described in detail at https://github.com/jan-brabec/microimaging_histology_DIB.

**Table 1:** Overview of histopathological classification of meningiomas samples. In total sixteen samples were collected and placed into the sample holder. The microstructural assessment adhered to the prevailing WHO criteria of 2016 as part of the clinical routine [15] since the data collection took place between the years 2016 and 2018.

Sample	WHO subtype	WHO grade
1	Transitional	I
2	Chordoid	II
3	Microcystic/Angiomatous	I
4	Meningothelial	II
5	Transitional	I
6	Meningothelial	II
7	Transitional	I
8	Meningothelial	I
9	Fibroblastic	I
10	Clear-cell	II
11	Transitional	I
12	Fibroblastic	I
13	Transitional	I
14	Microcystic/Angiomatous	I
15	Meningothelial	II
16	Transitional	I

**Table 2:** Diffusion-weighted measurements. With identical DTI protocol, the upper and lower part of the sample holder was imaged.

	Direction				
Measurement #	g_1_	g_2_	g_3_	Prescribed b-value [s/mm^2^]	Effective b-value [s/mm^2^]
1	0.58	0.58	0.58	100	71
2	0.30	-0.18	0.94	1000	1020
3	0.30	-0.14	0.94	3000	3008
4	-0.62	0.33	0.72	1000	1000
5	-0.62	0.33	0.72	3001	3001
6	-0.48	-0.70	0.53	1000	1000
7	-0.48	-0.70	0.53	3000	3001
8	0.97	0.16	0.21	1000	1037
9	0.98	0.12	0.18	3000	3017
10	0.29	0.82	0.50	1000	1000
11	0.29	0.82	0.50	3000	3001
12	0.50	-0.84	0.22	1000	1014
13	0.50	-0.84	0.19	3000	3000

**Table 3:** Description of raw and processed DTI measurements in the DTI_raw folder. The filename begins from the data path of the *data* folder.

Filename or data path	Description
DTI_raw/raw/nii	The directory contains raw NifTi files from the two DTI measurements. The lower part of the meningioma tumor sample holder (referred to as ‘down’) and the upper part of the holder (referred to as ‘up’).
DTI_raw/ROIs	The directory contains 16 regions of interest drawn around the slice that was coregistered (files ROI1.nii.gz, ROI2.nii.gz etc.).
DTI_raw/processed	The directory contains files processed by the DTI pipeline.

**Table 4:** Description of DTI and histology files of a single sample. Variable *s* indicates the sample number (1 to 16). The filename begins from the data path of the *data* folder and the data structure is the same for all sixteen individual samples.

File name or data path	Description
s/init_MR/ver1/MR.mat	Processed DTI maps of the MR slice further used for coregistration with histology are stored in the MR structure. Specifically, the MR structure contains S_0_, MD, FA, FA_IP_, AD, and RD parameters, and averaged DWI data across directions, all DWI data, and diffusion tensor eigenvalues in the x-y plane (J_11_, J_12_, J_22_).
s/raw_histo/HE.tif	Raw H&E-stained histology section in ‘.tif’ format.
s/raw_histo/EVG.tif	Raw EVG-stained histology section in ‘.tif’ format.
s/raw_histo/Metadata_HE.csv	Metadata acquired during the slide digitalization.
s/raw_histo/Metadata_EVG.csv	Metadata acquired during the slide digitalization.
s/raw_histo/HE_thumbnail.tif	Downsampled HE.tif file for an overview.
s/raw_histo/EVG_thumbnail.tif	Downsampled EVG.tif file for an overview.
s/coreg_rigid/HE.mat	H&E image stored as HE array that was approximately aligned with MR images from s/raw_MR/ver1/MR.mat.
s/coreg_rigid/EVG.mat	EVG image stored as EVG array that was approximately aligned with MR images from s/raw_MR/ver1/MR.mat.
s/coreg_fine/ver1/HE.mat	Coregistered H&E image stored as HE array.
s/coreg_fine/ver1/EVG.mat	Coregistered EVG image stored as EVG array.
s/coreg_fine/ver1/HE_mask.mat	Coregistered H&E mask (HE_mask), downsampled H&E mask to the MRI resolution (dHE_mask), and ROI (roi) structure.
s/coreg_fine/ver1/MR.mat	Coregistered MR structure of the processed DTI maps of the MR slice: S_0_, MD, FA, FA_IP_, AD, RD, averaged DWI data across directions, all DWI data, and J_11_, J_12_, J_22_.
s/coreg_fine/ver1/HE_lm_fine.mat	Landmark positions used for coregistration of MR to the H&E image stored in the lm_save structure.
s/coreg_fine/ver1/EVG_lm_fine.mat	Landmark positions used for coregistration of EVG to the H&E image stored in the lm_save structure.
s/coreg_fine/ver1/aniso2coreg.mat	Structure anisotropy (SA) map calculated for data/s/coreg_rigid/HE.mat to facilitate landmark definition based on FA_IP_ and SA.

**Fig. 1:**
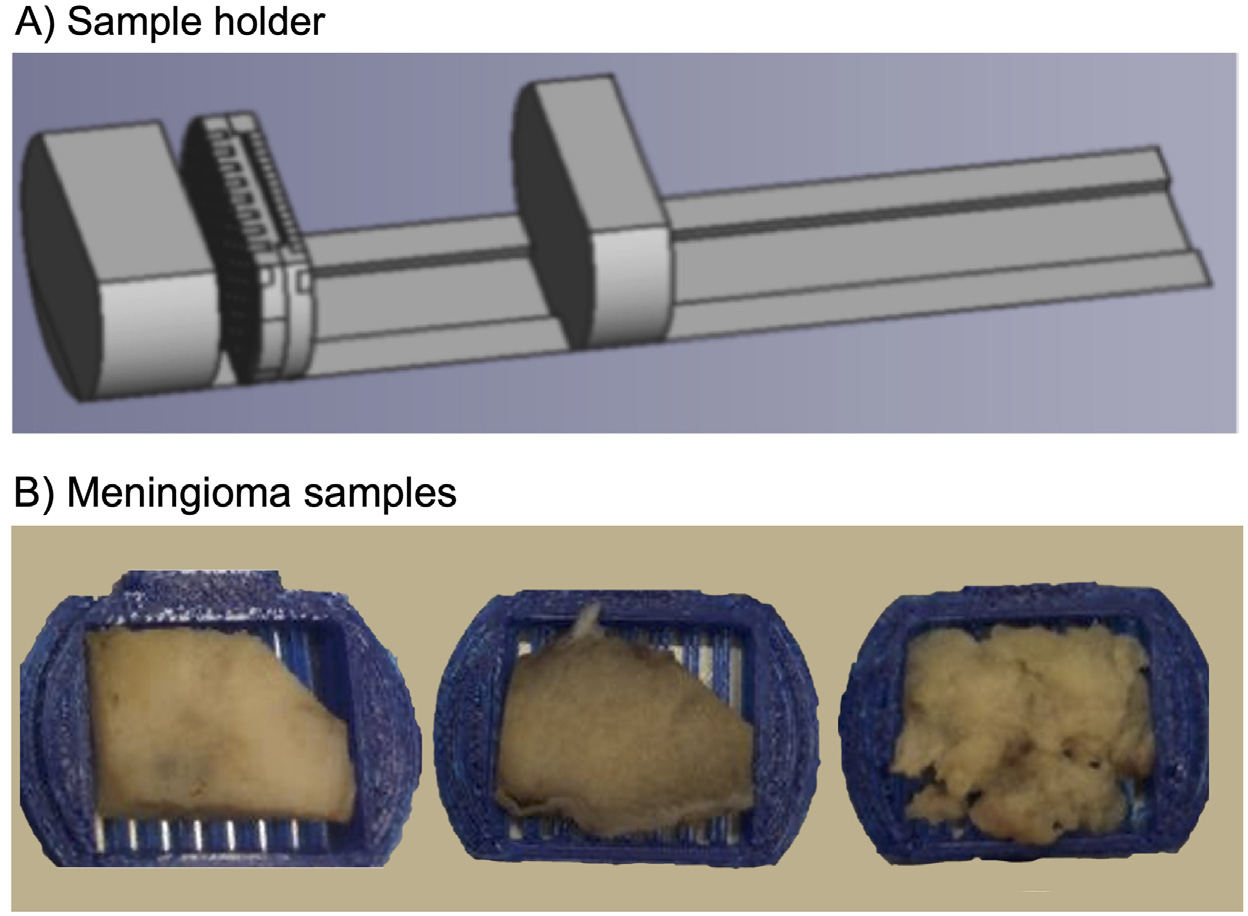
Panel A shows the sample holder. Panel B shows three examples of meningioma samples (left: 5, middle: 8, and right: 11). Panel A was adapted from Fig. 2A from a related research article [13], published by Elsevier and licensed under CC BY 4.0.

**Fig. 2:**
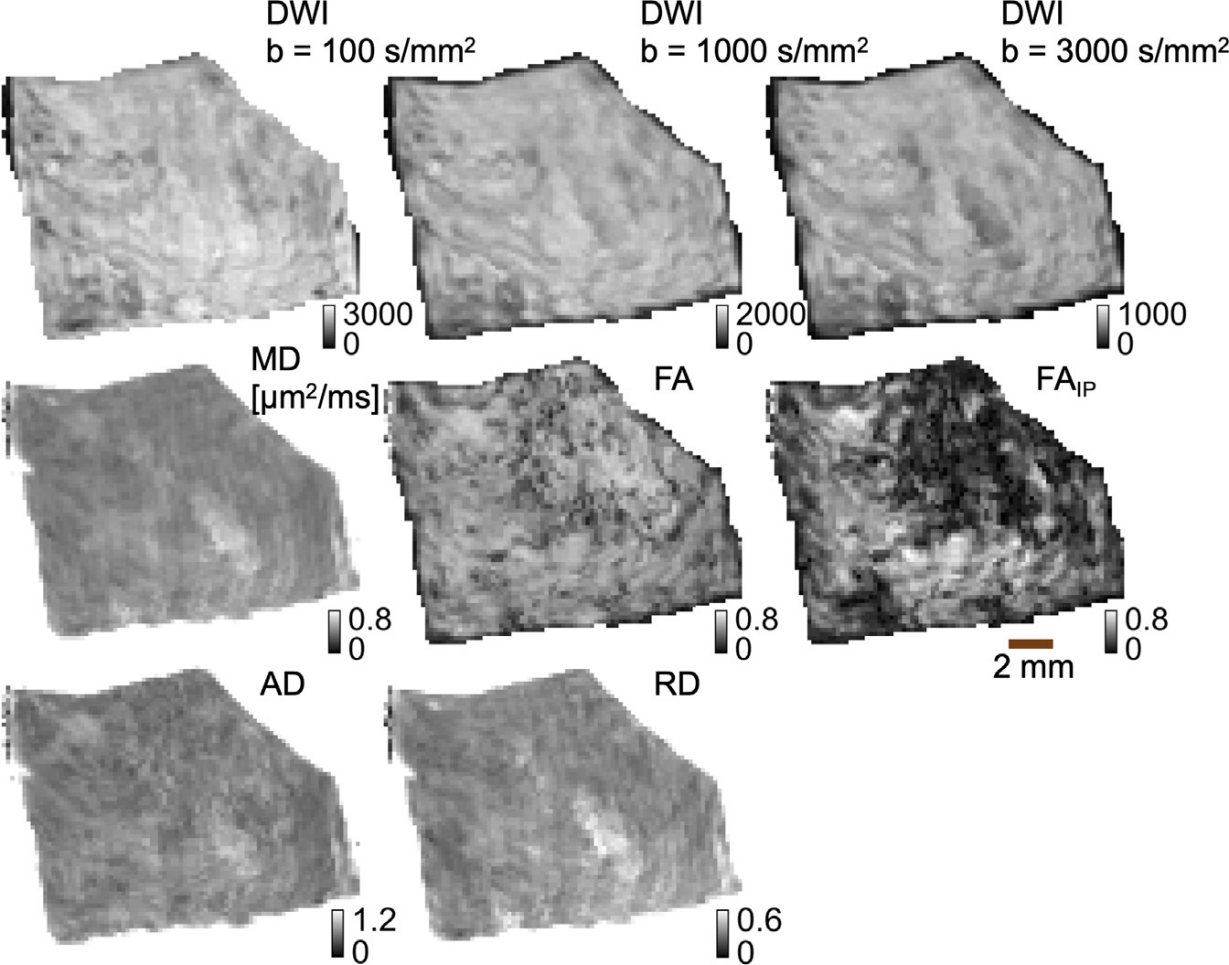
Overview of the DTI modalities in sample 5. In the upper row, the DWI signal averaged across all directions for the given b-value is shown. In the middle, row mean diffusivity (MD), fractional anisotropy (FA), and in-plane fractional anisotropy (FA_IP_) are visualized. In the bottom row, axial diffusivity (AD) and radial diffusivity (RD) are shown. Not shown are the eigenvalues of the diffusion tensor (J_11_, J_12_, J_22_).

**Fig. 3:**
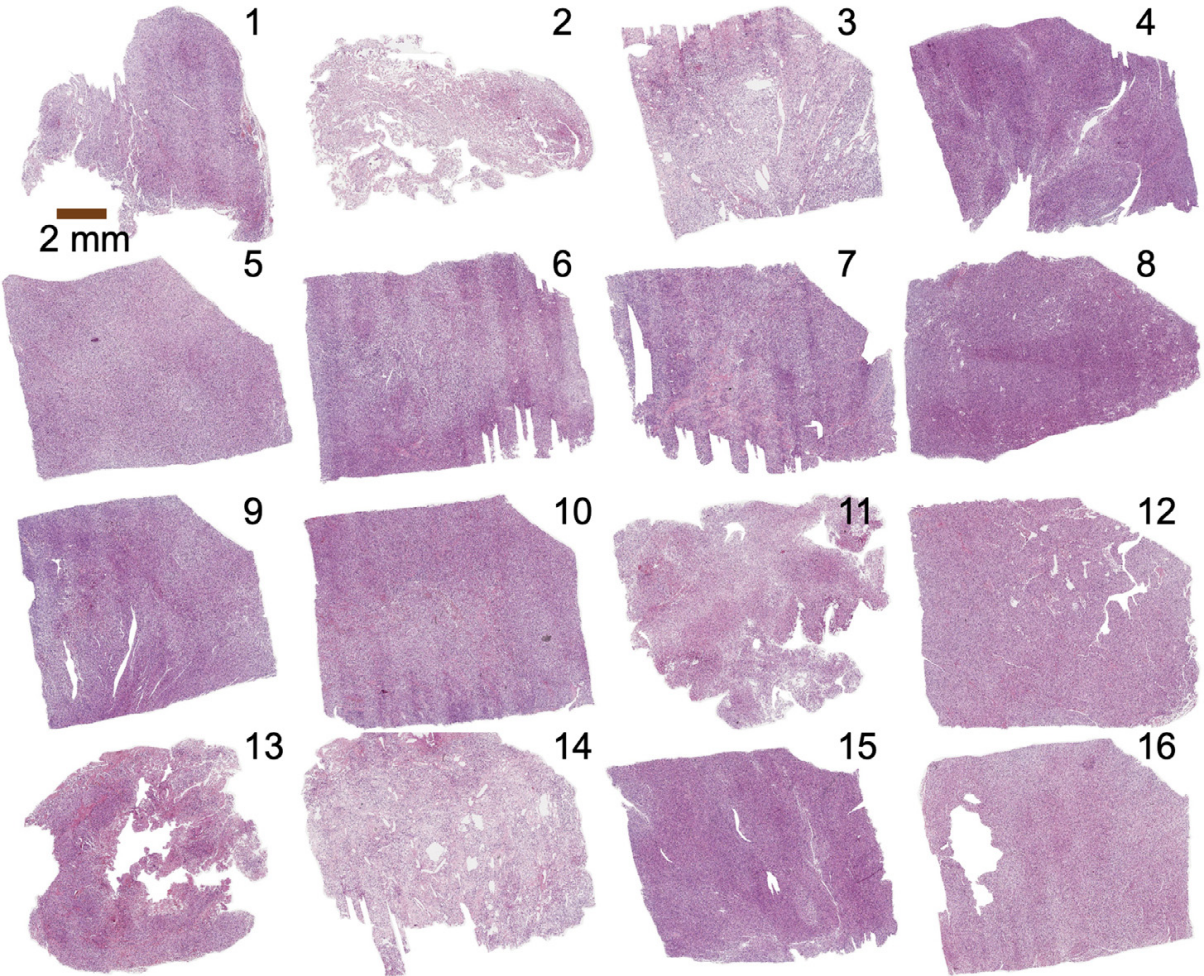
Overview of the H&E-stained meningioma histology sections of all samples. Sample number in the right upper corner. Figure adapted from Fig. 1A from related research article [13], published by Elsevier and licensed under CC BY 4.0.

**Fig. 4:**
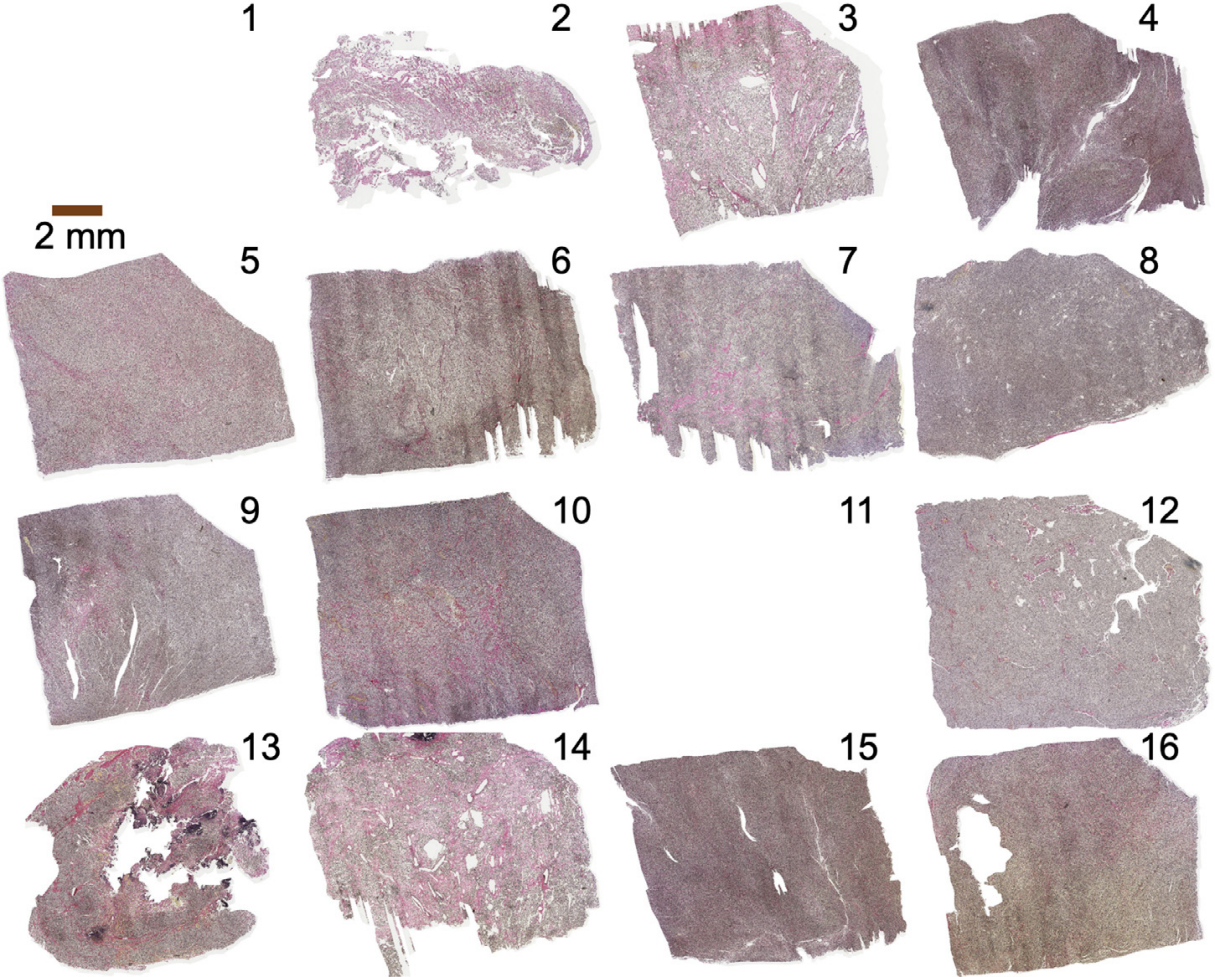
Overview of the EVG-stained meningioma histology sections of all samples except for 1 and 11. Sample number in the right upper corner.

**Fig. 5:**
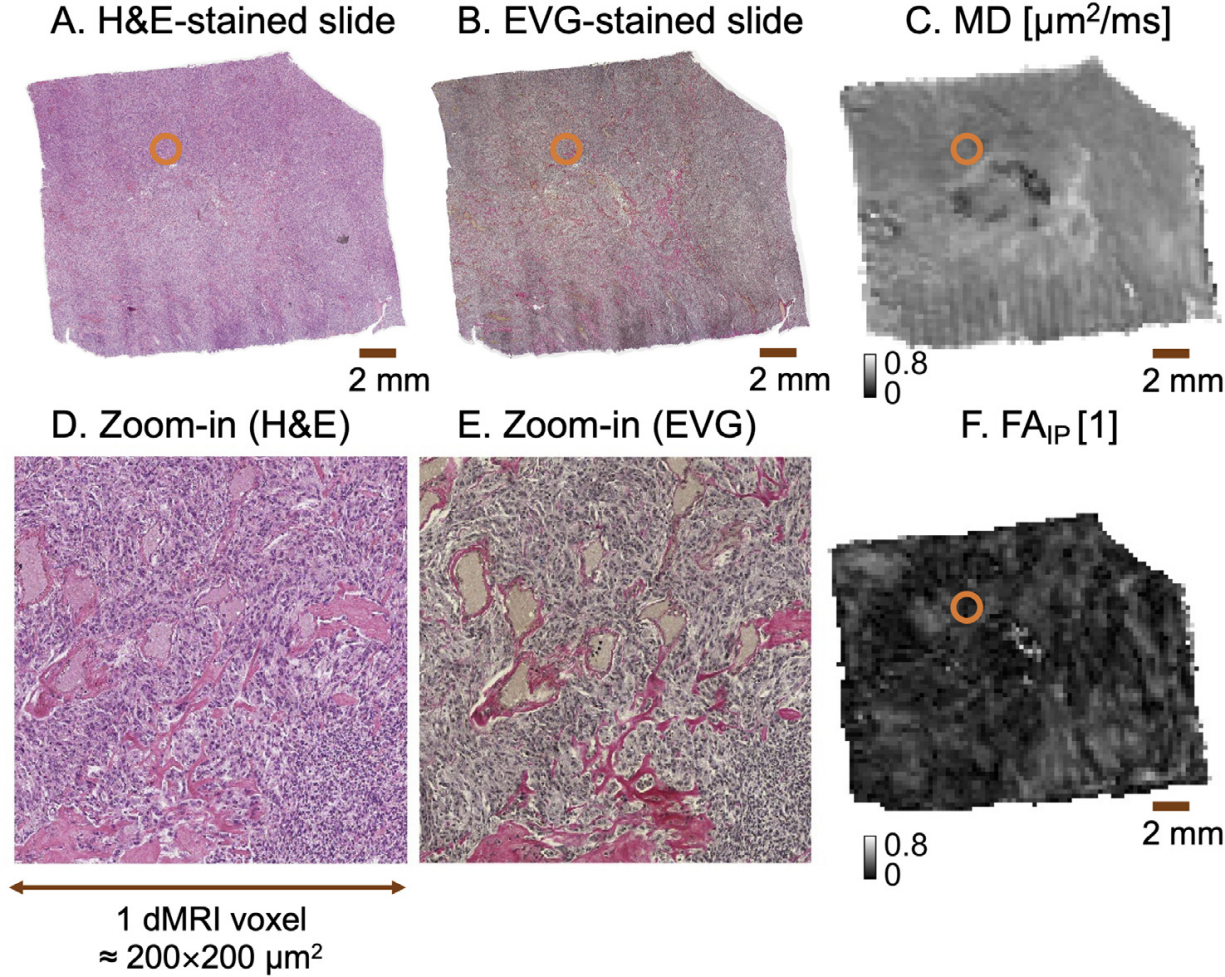
Overview of the coregistered data (sample 10). The figure shows a zoomed-out H&E- and EVG-stained histology sections panels A and B) with MD (panel C) and FA_IP_ (panel F). The orange circle exemplifies one particular voxel with zoomed-in H&E (panel D) and EVG-stained histology section (panel E). The figure also exemplifies the output of the function *view_HE_EVG_MR(sample)*.

The dataset from the AIDA repository is further illustrated by Figures. Fig. 1. shows an overview of the sample holder as well as examples of the meningioma samples. Fig. 2 shows the overview of the DTI modalities in a single sample. Figs. 3 and 4 show an overview of all meningioma histology sections stained with Hematoxylin and Eosin stain (H&E) and with Elastica van Gieson (EVG), respectively. Fig. 5 shows the overview of the coregistered data (sample 10).

Finally, the data structure from the AIDA repository concerning our related research article that is included in the same database is described in Table 5. These concern cell density obtained from QuPath software [16] and structure anisotropy maps. The details are available in the related research article [13] and the repository at https://github.com/jan-brabec/microimaging_vs_histology_in_meningeomas.

**Table 5:** Description of the files in our related research article. Variable *s* indicates the sample number (1 to 16). The filename begins from the data path of the *data* folder and the data structure is the same for all sixteen individual samples.

s/cell_density/QuPath	This directory contains a project folder with files automatically generated from QuPath software together with exported measurements (measurements.tsv) and QuPath readable image format (HE.jpg)
s/structure_anisotropy/ver1/SA.mat	Structure H contains the structure anisotropy (SA) map, spatial image derivatives (J_11_, J_12_, J_22_), downsampled SA (dSA), and downsampled spatial image derivatives (dJ_11_, dJ_12_, dJ_22_) to MR resolution.

## 3 Experimental Design, Materials and Methods

### 3.1 Patients and tumor specimens

This study included sixteen patients with diagnosed meningioma tumors scheduled for surgical treatment between 2016 and 2018 at Skåne University Hospital, Lund, Sweden. Inclusion criteria were age above 18 years, histopathologically confirmed meningioma, and signed informed consent. The study was approved by the Regional Swedish Ethical Review Authority, protocol number H15 642/2008 and 2018/37, and all subjects gave their written informed consent to participate in accordance with the Declaration of Helsinki. In total, sixteen tumor samples were obtained after neurosurgical excision and fixated in a formaldehyde solution (4%). Table 1 provides a summary of their histopathological evaluation and Fig. 1B example photographs of three distinct tumor specimens.

### 3.2 Data availability

The data are available at: https://www.doi.org/10.23698/aida/micromen and the processing code is available at: https://github.com/jan-brabec/microimaging_histology_DIB. A related research article with an example analysis is available in [13] and the code is at: https://github.com/jan-brabec/microimaging_vs_histology_in_meningeomas. The DTI measurements were processed using MATLAB version R2020a (The Mathworks, Natick, USA) by multidimensional diffusion MRI (MDM) framework available at: https://github.com/markus-nilsson/md-dmri [17].

### 3.3 Clinical histopathological evaluation

The meningioma tumor mass obtained from the neurosurgeons had been divided into two parts. Within the first part, each meningioma tumor specimen had been diagnosed by a histopathologist for tumor type and malignancy grade. This diagnostic procedure adhered to the prevailing WHO criteria of 2016 as part of the clinical routine [15] since the data collection took place between the years 2016 and 2018. The remaining part of the same tumor mass that was not needed for the clinical evaluation was used for our analysis.

### 3.4 DTI imaging

The tissue was cut into blocks of approximately 25 × 20 × 2 mm^3^ to fit a 3D-printed mold (Fig. 1A). Before the MR measurement, the tumor specimens were immersed in saline for a few hours to allow the water to diffuse throughout the tissue. The specimens were thereafter scanned at a Bruker 9.4 T BioSpec Avance III scanner. Diffusion-weighted imaging was performed using a spin-echo 3D-EPI DTI [3] sequence with TR = 1 s, TE = 30 ms, gradient duration δ = 4 ms, gradient separation Δ = 15 ms, effective diffusion time t_eff_ = (Δ – δ/3) = 13.6 ms, slices = 185, averages = 10, resolution=200 × 200 × 200 µm^3^, FOV = 27 × 27 × 37 mm^3^, image size = 139 × 139 × 185 pixels, fat suppression on, bandwidth 250 kHz, read offset = 0, phase offset = -2.615, slice offset = 0.101, coil RF RES 400 1H 059/035 QUAD TR, and with prescribed b-values of 100, 1000 and 3000 s/mm^2^ applied in six directions. The reason for choosing these b-values was that the average MD in the meningioma tumors was between 0.4 and 1.0 µm^2^/ms which translated to the optimal b-value between 1000 and 2500 s/mm^2^, assuming optimality when b·MD=1. To accommodate for regions with even lower MD we settled on a maximal b-value of 3000 s/mm^2^. Table 2 lists diffusion encoding direction and effective b-values that may be different from prescribed ones due to additional gradients used for spatial encoding. After the measurement, the tumor samples were again immersed in the formaldehyde fixative.

Since the tumor sample holder was larger than FOV, the MRI measurements were performed twice for the lower (referred to as ‘down’) and upper part of the sample (‘up’) with a total scan time of the two identical sequences of 6h 43m. These were non-overlapping except for sample number 8 which was imaged during both measurements but only the one from the ‘upper’ measurement was chosen for further analysis.

### 3.5 DTI processing

DICOM files were converted to compressed 4D NifTI (‘.nii.gz’) by dcm2niix (version 1.0.20220720) [18] available at https://github.com/rordenlab/dcm2niix. The b-tensor was obtained from the scanner and an appropriate experimental parameter structure (‘_xps.mat’) was constructed using the MDM framework [17]. We corrected the data for eddy-current-induced distortions by registering the images to an extrapolated reference [19] using ElastiX (version 5.0.1) [20]. These files are denoted with the suffix ‘_mc’. DTI analysis of the corrected images was performed with linear least squares fitting and the data smoothed with a Gaussian kernel with a size of 40 µm.

The following maps were obtained by a DTI analysis: S_0_ map (without diffusion encoding), mean diffusivity (MD), fractional anisotropy (FA), axial diffusivity (AD), radial diffusivity (RD). The eigenvalues J_11_, J_12_ = J_21,_ and J_33_ of the diffusion tensor **D** as well as the in-plane fractional anisotropy (FA_IP_) were also coregistered. FA_IP_ calculations are described in [13]. Furthermore, the orientationally averaged diffusion-weighted data at 100 s/mm^2^ (single measurement), 1000 s/mm^2^ (averaged across six directions), and 3000 s/mm^2^ (also averaged across six directions), as well as all individual diffusion-weighted data (thirteen measurements, Table 2) together with the obtained maps, were stored in the data structure ‘MR’ in ‘MR.mat’ in the folder ‘init_MR’. An overview of the modalities in a single sample is provided in Fig. 2.

### 3.6 Out-of-plane coregistration

Placing the specimens in a sample holder facilitated the coregistration with the histology sections, in particular the direction perpendicular to the tumor mass (out-of-plane). Because of the significant partial volume effects of the tumor with the sample holder in the first MR slice, the second MR slice was considered for further analysis. This also meant that the 200 µm of the tumor mass systematically needed to be removed on the same side of the sample holder before histopathological processing. The regions of interest (ROIs) were drawn around the whole second slice of the tumor sample and this region was used for coregistration.

### 3.7 Histopathological processing

The blocks on which DTI had been performed were embedded in paraffin, 200 µm of the mass removed (as described in the out-of-plane coregistration section), sectioned into 5 µm thin slices, and stained with Hematoxylin & Eosin (H&E) (overview in Fig. 3) and in the neighboring slice with Elastica van Gieson (EVG; Fig. 4). Sections were digitalized by a Hamamatsu NanoZoomer S360 digital slide scanner at a resolution of 0.5 × 0.5 µm^2^ and further metadata are saved in the files ‘Metadata_HE.csv’ and ‘Metadata_EVG.csv’. The raw histology images in .svs format were transformed to .tif images using ImageJ (https://imagej.nih.gov/ij/; version 1.53t) with the Bioformat plugin (https://docs.openmicroscopy.org/bio-formats; version 6.11.1) and the ‘.tif’ files are provided in our dataset.

### 3.8 In-plane landmark-based rigid coregistration of H&E- and EVG-stained sections to MRI

The in-plane coregistration accounted for different rotation angles between the MR slice and histology section as well as for the tissue deformation during the histology preparation. In this step, the H&E- and EVG-stained histology sections were rigidly rotated to approximately match the positions of the MR image. The rotation angle was calculated from landmarks’ positions placed on the edges of the histology section and the MR of the tumor sample. The cropping points were then manually defined outside of the tumor sample and the resulting images were stored in the folder ‘coreg_rigid’ as ‘.mat -v7.3’ files. These can be opened using MATLAB, Octave, or Python using the mat73 module. The reason why we settled on using this file format was that it provided superior writing and reading speed for the large files compared with the ‘.tif’ format in the MATLAB environment.

### 3.9 In-plane landmark-based deformable coregistration of MR to H&E-stained histology sections

After approximate alignment, the MR images were coregistered to H&E-stained histology sections by a non-linear landmark-based approach. The landmarks were defined on the MD and FA_IP_ maps and then on the corresponding structures in the histology sections. Landmarks were placed at the corners and edges of the sections and also in tumor microscopic features, such as tumor microvasculature, readily discernible in both the histology sections and MR images. The resulting images were stored in the data folder in the ‘coreg_fine’ folder together with defined landmarks and mask around the H&E-stained image.

### 3.10 Landmark-based rigid coregistration of EVG- to H&E-stained histology sections

EVG-stained histology sections were coregistered rigidly to the H&E-stained histology sections based on a landmark-based approach. These were defined in both sections in the same microstructural features and the images were stored in the ‘coreg_fine‘ folder. The EVG-stained images of samples 2 and 11 are missing because the files were corrupted during the slide digitalization.

Finally, we investigated qualitatively the coregistration accuracy with a script that is located in the folder ‘Step_5_View_data’ and named ‘view_HE_EVG_MR(sample)’ (Fig. 5).

## Ethics Statements

This study included sixteen patients with radiologically diagnosed meningioma tumors scheduled for surgical treatment between 2016 and 2018 at Skåne University Hospital, Lund, Sweden. Inclusion criteria were age above 18 years, histologically confirmed meningioma, and signed informed consent. The study was approved by the Regional Swedish Ethical Review Authority, protocol number H15 642/2008 and 2018/37, and all subjects gave their written informed consent to participate in accordance with the Declaration of Helsinki.

## CRediT Author Statement

**Jan Brabec:** Conceptualization, Methodology, Formal analysis, Investigation, Visualization, Data Curation, Writing – Original Draft, Writing – review & Editing **Elisabet Englund:** Resources, Investigation, Project administration, Writing – review & Editing **Johan Bengzon:** Resources, Funding acquisition, Project administration, Writing – review & Editing **Filip Szczepankiewicz:** Conceptualization, Methodology, Software, Supervision, Writing – Original Draft, Writing – review & Editing **Danielle van Westen:** Funding acquisition, Project administration, Writing – review & Editing, Ethics application **Pia C Sundgren:** Funding acquisition, Project administration, Writing – review & Editing **Markus Nilsson:** Conceptualization, Funding acquisition, Visualization, Methodology, Software, Project administration, Supervision, Writing – review & Editing.

## Declaration of Competing Interest

The authors declare that they have no known competing financial interests or personal relationships that could have appeared to influence the work reported in this paper.

## Data Availability

Data (Original data) (MicroMen dataset hosted by AIDA data hub registry). Supporting Code (Original data) (Microimaging vs histology meningiomas DIB repository hosted by GitHub).
